# Effect of Temperature on Acetate Mineralization Kinetics and Microbial Community Composition in a Hydrocarbon-Affected Microbial Community During a Shift From Oxic to Sulfidogenic Conditions

**DOI:** 10.3389/fmicb.2020.606565

**Published:** 2020-12-17

**Authors:** Mohammad Sufian Bin Hudari, Carsten Vogt, Hans Hermann Richnow

**Affiliations:** Department of Isotope Biogeochemistry, Helmholtz Centre for Environmental Research, Leipzig, Germany

**Keywords:** aquifer thermal energy storage, ATES, bioremediation, microbial community, acetate, sulfate reduction

## Abstract

Aquifer thermal energy storage (ATES) allows for the seasonal storage and extraction of heat in the subsurface thus reducing reliance on fossil fuels and supporting decarbonization of the heating and cooling sector. However, the impacts of higher temperatures toward biodiversity and ecosystem services in the subsurface environment remain unclear. Here, we conducted a laboratory microcosm study comprising a hydrocarbon-degrading microbial community from a sulfidic hydrocarbon-contaminated aquifer spiked with ^13^C-labeled acetate and incubated at temperatures between 12 and 80°C to evaluate (i) the extent and rates of acetate mineralization and (ii) the resultant temperature-induced shifts in the microbial community structure. We observed biphasic mineralization curves at 12, 25, 38, and 45°C, arising from immediate and fast aerobic mineralization due to an initial oxygen exposure, followed by slower mineralization at sulfidogenic conditions. At 60°C and several replicates at 45°C, acetate was only aerobically mineralized. At 80°C, no mineralization was observed within 178 days. Rates of acetate mineralization coupled to sulfate reduction at 25 and 38°C were six times faster than at 12°C. Distinct microbial communities developed in oxic and strictly anoxic phases of mineralization as well as at different temperatures. Members of the Alphaproteobacteria were dominant in the oxic mineralization phase at 12–38°C, succeeded by a more diverse community in the anoxic phase composed of Deltaproteobacteria, Clostridia, Spirochaetia, Gammaproteobacteria and Anaerolinea, with varying abundances dependent on the temperature. In the oxic phases at 45 and 60°C, phylotypes affiliated to spore-forming Bacilli developed. In conclusion, temperatures up to 38°C allowed aerobic and anaerobic acetate mineralization albeit at varying rates, while mineralization occurred mainly aerobically between 45 and 60°C; thermophilic sulfate reducers being active at temperatures > 45°C were not detected. Hence, temperature may affect dissolved organic carbon mineralization rates in ATES while the variability in the microbial community composition during the transition from micro-oxic to sulfidogenic conditions highlights the crucial role of electron acceptor availability when combining ATES with bioremediation.

## Introduction

Aquifer thermal energy storage (ATES), leveraging on seasonal heat storage and extraction, promises a more sustainable alternative to reduce fossil fuels dependence for energy supply, especially in the heating and cooling sector (Elsland et al., 2017). For example, this sector serves residential and industrial consumers by providing solutions to energy demands for space or process heating and cooling. ATES allows for the storage of excess thermal energy during the warm summer to meet higher thermal energy demands in the cooler seasons. ATES operating temperatures vary from one installation to another (Fleuchaus et al., 2018), although they typically function under an upper temperature limit as regulatory threshold, e.g., 25°C, adopted by many Low-Temperature ATES (LT-ATES) operations (Drijver et al., 2012). Operating above this temperature threshold, e.g., up to 60°C and higher, as in the case of High Temperature ATES (HT-ATES), has since garnered interest as authorities sought to diversify their sources of energy supply. Sites where ATES systems are situated are usually urban or industry areas which are frequently loaded with contaminants released by former anthropogenic activities and thus limited as resources for drinking water. Contaminants commonly found in urban aquifers consist of petroleum hydrocarbons introduced via spillage or improper disposal (Rooney-Varga et al., 1999; Da Silva and Alvarez, 2004; Vogt et al., 2007), where their persistence remains a big hurdle to remediation up to today (NARA, 2018). In the subsurface, microbial biodegradation is crucial since contaminants can be mineralized to harmless products (e.g., carbon dioxide and water), whereas remediation techniques based on abiotic processes are usually limited in their ability to mineralize contaminants (EPA, 1999). Most hydrocarbons are biodegradable at oxic and anoxic conditions, in aquifers, however, anaerobic biodegradation is the more important attenuation process since conditions turn rapidly anoxic once oxygen is consumed. Anaerobic hydrocarbon degradation pathways are well-known and are usually carried out by microbial consortia (Mancini et al., 2002; McKelvie et al., 2005; Gieg et al., 2010; Winderl et al., 2010; Fuchs et al., 2011; Meckenstock et al., 2015; Lueders, 2017). Acetate is a key intermediate especially during syntrophic hydrocarbon biodegradation. It serves a dual role as an electron donor and a soluble carbon source which can be easily transported and utilized by microorganisms (Wu et al., 1997; Ho et al., 2002; Heuer et al., 2010; Head et al., 2014), both aerobes (Wu et al., 1997) and anaerobes (Sansone and Martens, 1982; Schink and Stams, 2006; Heuer et al., 2010). At anoxic conditions, sulfate is a main electron acceptor in marine but also freshwater habitats making dissimilatory sulfate reduction a commonplace in contaminant degradation (National Research Council, 2000; Miao et al., 2012).

Generally, biodegradation processes accelerate at higher temperature leading to the hypothesis that organic contaminants may be preferentially degraded at elevated temperatures during ATES operation, enabling the possibility to combine *in situ* bioremediation and ATES. However, unlike Arrhenius-like models where biochemical rates tend to increase with temperature (Mohr and Krawiec, 1980; Huang et al., 2011), the temperature-microbial growth paradigm is somewhat different, i.e., species are categorized according to their maxima, minima, and optimal growth temperatures (Dettmer, 2002). In general, growth ranges of psychrophiles, mesophiles, thermophiles and hyperthermophiles are: 0–20, 8–48, 40–70, and 65–90°C, respectively (Madigan et al., 2017). Optimal temperatures allow maximal growth rates; rates will be significantly lower and finally cease when temperatures reach sub-optimal and boundary conditions. This is paramount since ATES are likely to drive seasonal temperature fluctuations in the subsurface. As such, temperature fluctuations from ATES could influence the structure of the indigenous microbial community, its biodegradation potential, as well as the physicochemical characteristics of the contaminants such as adsorption and solubility (Koproch et al., 2019).

In ATES, it is postulated that while temperatures < 40°C may enhance biodegradation by increasing microbial activities of mesophiles, temperatures as high as 80°C or above may considerably affect the structure and function of subsurface microbial communities and adversely impact biodegradation. Nonetheless, the mineralization capacity of the indigenous microbial community is imperative in a comprehensive assessment of combined ATES and remediation potential. While the effects of temperature on the degradation of hydrocarbons and its key intermediate acetate have been widely reported, temperatures are not specifically geared toward ATES-related and microbial growth temperature targets. Moreover, not much has been reported on microbial community shifts during the transition from an oxic to an anoxic environment. Henceforth, in this study, the effects of temperature on (i) extent and rates of acetate mineralization by a hydrocarbon-adapted microbial community at ATES-relevant temperatures and (ii) temperature-induced shifts in the microbial community structure are addressed.

## Materials and Methods

### Chemicals

All chemicals used in this study were of pro analysis quality and purchased from Sigma-Aldrich (United States), Merck (Germany) and Carl Roth (Germany) unless stated otherwise. Uniformly ^13^C-labeled sodium acetate was purchased from Cambridge Isotope Laboratories Inc. (98% purity, 99 atom%, Massachusetts, United States). *N,N*-dimethyl-1,4-diphenylenediammonium dichloride (DMPD) was obtained from Merck (Switzerland).

### Experimental Setup

For our experiment, we used coarse sand taken from columns of an *on-site* system percolated with sulfidic benzene-contaminated groundwater for 10 years and 6 months at a mean temperature of 15°C as described elsewhere (Vogt et al., 2007; Taubert et al., 2012). The coarse sand was collected in January 2017 and maintained since then in 1 L Schott bottles filled with anoxic bicarbonate-buffered mineral salt media comprising 10 mM sulfate as electron acceptor and supplemented with 10 μL of benzene dissolved in 15 mL of the inert 2,2,4,4,6,8,8-heptamethylnonane (HMN) as a carrier phase. These bottles were incubated at room temperature (∼20°C) and were re-supplemented with benzene and sulfate if substrates had been consumed. For this study, microcosms were prepared in October 2018 using the coarse sand material from one of the bottles in an anaerobic glove box (95% N_2_/5% H_2_ in normal operation, Coy Laboratory Products, United States). Each microcosm comprised of 40 g of gravel and 80 mL of modified mineral salt medium (Bak and Pfennig, 1991) in 120 mL serum bottles which were gas-tight sealed with Teflon-coated butyl rubber stoppers (Wheaton Industries, United States) and crimped aluminum caps. The recipe of the modified mineral salt medium is described in the Supplementary Table S1.

A total of 65 replicate bottles were set up in the glove box distributed amongst six temperature settings: 12°C (11 bottles), 25°C (12 bottles), 38°C (12 bottles), 45°C (12 bottles), 60°C (11 bottles), and 80°C (7 bottles). Oxygen was accidentally introduced during this step arising from a technical incident and a faulty oxygen sensor. For each incubation temperature, three bottles were used as replicates for analytical measurements during the whole incubation period and two for sterile controls for the same purpose. The remaining replicates were sacrificed at various time points of the incubation period for microbial community analysis. Replicates incubated at 12, 25, 38, and 45°C were finally sacrificed when acetate mineralization was completed based on sulfide and δ^13^CO_2_ data. Prior to sodium acetate amendments, all microcosms were incubated at 12, 25, 38, 45, 60, and 80°C for 3 days to guarantee that microcosm had reached the experimental temperatures before acetate addition. All replicates were amended with uniformly ^13^C-labeled sodium acetate by a plastic syringe (B Braun, Germany) to a final concentration of 1 mM from a pre-prepared sterile anoxic stock solution (100 mM), except for two replicates at 12°C (sacrificed on days 214 and 220) which were further maintained by the addition of 750 μL of the stock solution of ^13^C-labeled acetate (100 mM). Microcosms were incubated horizontally and statically in the dark at 12, 25, 38, 45, 60, or 80°C using various incubators (IN55, Memmert, Germany; Heraeus incubator, Thermoscience, United States). Samples and headspace collection vials (10 mL) for chemical analyses over the course of the experiment were conducted by means of sterile, nitrogen-flushed syringes. Headspace collection vials were sealed with Teflon-coated butyl rubber stoppers and aluminum crimps. All used glassware was sterilized by autoclaving (121°C, 20 min) before usage.

### Chemical Analyses

#### Analysis of δ^13^CO_2_

Headspace samples (4 mL) were collected over the course of the experiment using N_2_-flushed syringes into N_2_-flushed (2 min, 1 bar) collection vials (10 mL) which were sealed with Teflon-coated butyl rubber stoppers and aluminum crimps. The carbon isotope ratios of headspace CO_2_ and CH_4_ were measured by gas chromatography-combustion-isotopic ratio mass spectrometry (GC-IRMS) comprising a gas chromatograph (Agilent, United States) coupled to a mass spectrometer via a Conflow III Interface (Germany). The column used for the measurement was a PoraplotQ column (25 m × 0.32 mm ID, 1 μm film; Chrompack, Middleburg, United States). Carbon dioxide separation was achieved isothermally at 40°C using helium as the carrier gas at a flow rate of 2 mL min^–1^, with an expected elution time of 500 s after injection. After the separation, the sample was transferred to a combustion furnace (GC/C-III; Thermo Fisher Scientific, Bremen, Germany) held at 980°C on a CuO/Ni/Pt catalyst to track also carbon isotope signatures of methane which would have been converted to carbon dioxide. However, methane was never detected in any of the measured samples. Samples of different volumes (100–500 μL) were injected at 1:5 split ratios. Carbon isotope signatures are given in delta notation (per mill, ‰) relative to the Pee Dee Belemnite standard.

The percent of mineralization (MIN%) of each replicate was calculated according to the following equation (Dorer et al., 2016):

(1) $$MIN\left(\%\right)=\frac{\left[H\,C\,O_{3}^{-}\right]\times R_{V\,P\,D\,B}\times \left(\delta_{e\,n\,d}-\delta_{s\,t\,a\,r\,t}\right)}{\left(n\right)\times \left[a\,c\,e\,t\,a\,t\,e\right]\times \left(1+R_{V\,P\,D\,B}\times \left(1+\delta_{\left(s\,t\,a\,r\,t\right)}\right)\right)}\times 100$$

This takes into account the starting (δ_start_) and endpoint (δ_end_) isotope values (‰), bicarbonate (HCO${}_{3}^{-}$) and acetate concentration and number of labeled carbon of the target compound, where in the case of fully labeled acetate, *n* = 2. This allows a relative comparison of the extent of acetate mineralization.

#### Analysis of Sulfide

Sulfide was quantified in liquid samples using the modified methylene blue method originally described elsewhere (Cline, 1969; Muller et al., 2009). Samples (200 μL) were collected over the course of the experiments via sterile N_2_-flushed plastic syringes and immediately added to 1 mL of 3% zinc acetate dihydrate (Merck, Germany) in glass test tubes. Four mL of bi-distilled water was added followed by the addition of 400 μL of Cline reagent, which was prepared in an amber Schott bottle by dissolving (per 50 mL) 1 g of *N,N*-dimethyl-1,4,-diphenylenediammonium dichloride (Merck, Switzerland) and 1.5 g FeCl_3_ × H_2_O (Honeywell, United States) in 25% HCl (Merck, Germany). The mixture was incubated in the dark for 20 min, subsequently vortexed and transferred to 2.5 mL cuvettes in which the absorbance at 670 nm was determined with a spectrophotometer (Novaspec III, Amersham Biosciences, Cambridge, United Kingdom) using samples prepared with bi-distilled water as a blank.

### Microbial Community Analysis

Microcosms were sacrificed randomly over a time course from each treatment temperature. Sessile cells were harvested from the coarse sand material by a series of sodium tetrapyrophosphate treatment using a modified method described by Velji and Albright (1984). Firstly, 0.6 mL of cold, sterile 1% sodium tetrapyrophosphate (Sigma–Aldrich, United States) was added to each microcosm bottle to give an approximate final concentration of 0.01% sodium tetrapyrophosphate; the mixture was sonicated for 1 min in a sonicating bath (Bandelin, Germany). The liquid was transferred into a new 50 mL Falcon tube. The step was repeated at least three times: two rounds of sonication with 5 mL of 1% sodium tetrapyrophosphate for 1 min followed by a final round of 5 mL of 1% sonication for five min. After each round of sonication, the liquid was transferred into a Falcon tube and kept cool in an ice bath. At the end of the sonication step, the Falcon tubes were centrifuged at 11.000 rpm at 4°C (Type 5804R, Eppendorf, Germany). The supernatant was discarded and the pellet was stored at −20°C in the freezer until DNA extraction. DNA was extracted from thawed cell-sand debris pellets with the DNeasy PowerSoil Kit (Qiagen, Hilden, Germany) following the manufacturer’s instructions. DNA was eluted in 40 μL elution buffer. Extracted DNA was quantified using a Qubit 3.0 Fluorometer (Life Technologies, Malaysia) with the Qubit HS (High Sensitivity) Assay kit (Thermo Fisher Scientific, United States).

16S rRNA genes were amplified using the Klindsworth primer pair (S-D-Bact-0341-b-S-17/S-D-Bact-0785-a-A-21) generating an amplicon of 464 bp (Klindworth et al., 2013). The PCR was carried out according to the following protocol (preparation and program) in an S1000 Thermal Cycler (Bio-Rad, United States). Each PCR reaction mixture (25 μL volume) consisted of 12.5 μL of MyTaq (Bioline), 1 μL of each primer 341F/785R (10 pM), 1 μL of Bovine Serum Albumin (1:20) and up to 15 ng of DNA, quantified via the Qubit 3.0 Fluorometer. The PCR program based on 10–15 ng of DNA comprised of an initial activation step of 95°C for 3 min, 25 cycles of: 30 s at 95°C for denaturation, 30 s at 55°C for annealing, 30 s at 72°C, and a final extension step at 72°C for 5 min. For DNA samples containing less than 10 ng, 30 or 35 cycles were used instead of 25. The 16S PCR products were verified via gel electrophoresis (1.5% agarose) and purified with Ampure XP beads (ThermoFisher, United States), and verified again via gel electrophoresis. Sequencing libraries were prepared using the Illumina MiSeq Reagent Kit v3 (2 × 300 bp) as recommended by the manufacturer on 16S Metagenomic Sequencing Library Preparations (Illumina, 2013). Briefly, multiplex indexing barcodes were added using Illumina Nextera Xt Index Kit, subsequently purified by Ampure XP beads; the libraries were subsequently quantified with the Qubit BR (Broad Range) Assay kit (Thermo Fisher Scientific, United States). Purified PCR products were each diluted to 4 nM and pooled together. The pooled libraries were sequenced on an Illumina Miseq platform at the Department of Environmental Microbiology of the Helmholtz Centre for Environmental Research –UFZ. The sequences were analyzed using the QIIME 2 v2019.1 (Bolyen et al., 2019) pipeline provided by Dr. Denny Popp (UFZ, Department of Environmental Microbiology, Systems Biology of Microbial Communities Working Group). Briefly, primer sequences and adapters appended during the library preparation were removed from the de-multiplexed sequences using Cutadapt v1.18 (Martin, 2011). Sequences were further trimmed, denoised by removing low quality reads and chimeras and merged using DADA2 (Callahan et al., 2016). Amplicon sequence variants (ASVs) were assigned to bacterial DNA against the silva132 database (Quast et al., 2013; Yilmaz et al., 2014). Finally, rarefaction curves and diversity indices (Bray–Curtis Dissimilarity) were generated using the QIIME 2 software to assess if the sampling depth was sufficient and to test if there are significant differences between the incubation conditions toward the microbial community composition. For analysis of Bray–Curtis dissimilarity, samples were rarefied to the minimum sequencing read with minor losses to the total amplicon sequencing variants ASVs (Supplementary Figure S1). Sequences were deposited at the European Nucleotide Archive (ENA) under the primary accession number PRJEB40338.

## Results

### Effect of Temperature on Acetate Mineralization

**FIGURE 1:**
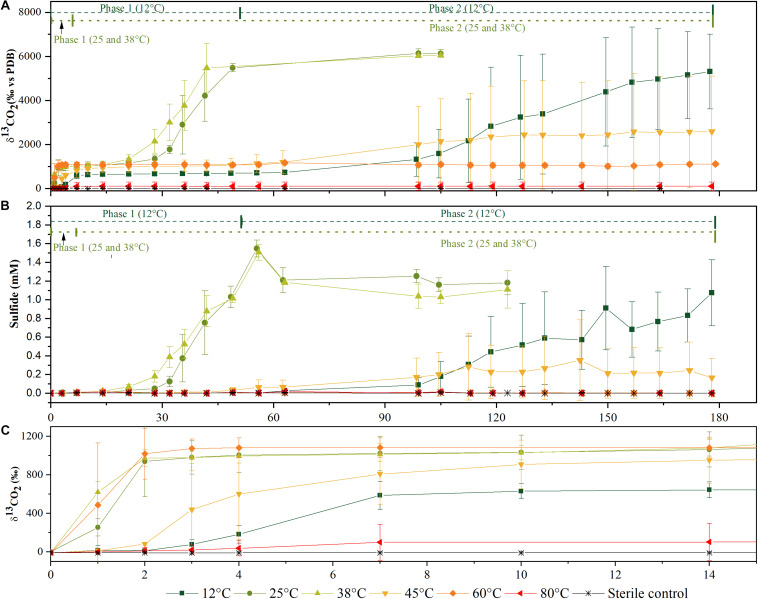
Time courses of ^13^C-labeled CO_2_ production **(A)** and of sulfide production **(B)** from microcosms incubated at different temperatures in the aerobic phase 1 and anaerobic phase 2 of mineralization. Zoom in of the time course during the first 15 days of **(A,B)** for ^13^C-labeled CO_2_ production **(C)** for higher resolution of mineralization phase 1. Data represented as an average of three biological replicates. Error bars represent ± standard deviations of the triplicate values.

Acetate was mineralized at all temperatures, at varying degrees and different rates. In Figures 1A,B, data for δ^13^CO_2_ and sulfide concentrations for the six different temperatures are shown. These values were derived from triplicate cultures, each incubation temperature was set up with at least eight replicates which were sacrificed for biodiversity analysis at different time points. The δ^13^CO_2_ composition and sulfide concentration were monitored for all replicates until they were sacrificed. These data were used to derive the percentage of mineralization and concentrations of acetate utilized and sulfide produced (Table 1). Generally, δ^13^CO_2_ and sulfide values of all replicates behaved similarly to the data of the triplicates shown in Figure 1 within each temperature setting unless stated otherwise. Phase 1 is characterized by the evolution of ^13^CO_2_ until a plateau was reached within 7–28 days (Figure 1C and Table 1). Increases of δ^13^CO_2_ values due to ^13^CO_2_ formation by ^13^C_2_-acetate oxidation were observed at all temperatures within 24–48 h (data not shown), illustrating an immediate begin of acetate turnover. At 80°C, only one replicate mineralized acetate, albeit in a limited way, as observed during the first 7 days of incubation (Table 1). Above all, this phase was considered as a putative aerobic phase since no sulfide was detected in this period.

**TABLE 1:** Overview on acetate mineralization, sulfide production and theoretical oxygen consumed in individual experiments.

**Temp′**	**Replicate**	**Phase**	**Period (*d*)**	**Duration (*d*)**	**Acetate mineralization**				**Sulfide production**			Theoretical oxygen consumed*	
							
					**Mineralization (%)**	**Acetate mineralized (μM)**	**Rate (μM/d)**	**Average (μM/d)**	**Sulfide produced (μM)**	**Rate (μM/d)**	**Average (μM/d)**	**Concentration (μM)**	**Average (μM)**
12°C	1		0–28	28	12.0	119.8	4.3			–		239.6	
	2	1	0–7	7	11.8	118.2	16.9	12.2 ± 6.9	Not observed	–	–	236.3	231.2 ± 11.8
	3		0–7	7	10.9	108.8	15.5			–		217.6	
	
	1		63–127	64	93.1	931.0	14.5		860.0	13.4		–	
	2	2	63–164	101	86.7	867.2	8.6	9.8 ± 4.3	725.5	7.2	8.75 ± 4.1	–	–
	3		63–214	151	94.1	941.0	6.2		848.7	5.6		–	

25°C	1		0–2	2	13.8	138.2	69.1			–		276.3	
	2	1	0–2	2	22.4	223.8	111.9	75.0 ± 34.4	Not observed	–	–	447.7	358.5 ± 85.9
	3		0–2	4	17.6	175.8	43.9			–		351.6	
	
	1		10–49	39	80.2	801.5	20.6		850.9	24.3**¶**		–	
	2	2	10–49	39	70.3	703.2	18.0	18.8 ± 1.5	637.0	18.2**¶**	21.26 ± 3.1	–	–
	3		10–49	39	69.7	696.6	17.9		744.8	21.3**¶**		–	

38°C	1		0–2	2	17.6	176.1	88.1			–		352.2	
	2	1	0–3	3	15.3	152.8	50.9	74.1 ± 20.2	Not observed	–	–	305.5	330.5 ± 23.5
	3		0–2	2	16.7	166.9	83.4			–		333.7	
	
	1		10–42	32	81.2	811.7	25.4		686.4	19.6§		–	
	2	2	10–42	32	53.6	536.1	16.8	22.9 ± 5.3	447.3	12.8§	18.04 ± 4.7	–	–
	3		10–42	32	84.8	848.0	26.5		760.2	21.7§		–	

45°C	1		0–21	21	13.7	137.1	6.5			–		274.3	
	2	1	0–28	28	15.2	151.7	5.4	10.7 ± 8.2^†^ (*n* = 3)	Not observed	–	–	303.4	326.7 ± 67.2
	3		0–10	10	20.1	201.3	20.1	6.0 ± 0.8^‡^ (*n* = 2)		–		402.5	
	
	1		–	–	–		–		–	–		–	
	2	2	–	–	–		–	7.6	–	–	5.4	–	–
	3		42–127	85	64.7	647.1	7.6	(*n* = 1)	461.8	5.4	(*n* = 1)	–	

60°C	1		0–3	3	18.0	180.5	60.2			–		361.0	
	2	1	0–3	3	16.4	164.0	54.7	71.4 ± 24.3	Not observed	–	–	328.0	362.0 ± 34.5
	3		0–2	2	19.9	198.5	99.3			–		397.1	
	
	1												
	2	2	Not observed	–	–	–	–	–	Not observed	–	–	–	–
	3												

80°C	1		0–7	7	5.4	53.8	7.7			–		107.5	
	2	1	–	–	–	–	–	7.7	Not observed	–	–	–	107.5
	3		–	–	–	–	–	(*n* = 1)		–		–	(*n* = 1)
	
	1												
	2	2	Not observed	–	–	–		–	Not observed	–	–	–	–
	3												

*The theoretical oxygen consumption was calculated according to the equation: CH_3_COOH + 2 O_2_ → 2CO_2_ + 2 H_2_O. **¶**, § No sulfide data available for day 10. Sulfide production data and rates calculated between periods 14–49 and 7–42 for 25 and 38°C respectively. †, ‡Calculation with/without inclusion of replicate 45°C-3 value.

Rates of acetate mineralization were calculated to compare between the short burst of activity in the putatively aerobic phase 1 and the prolonged anaerobic phase 2 which are represented in Table 1. Phase 1 mineralization rates were found to be similar at 25°C (75.0 ± 34.3 μM d^–1^), 38°C (74.1 ± 20.2 μM d^–1^), and 60°C (71.4 ± 24.3 μM d^–1^), with no apparent lag phase (Table 1). The degree of mineralization was also similar at 60°C (16.4–19.8% mineralized), 25°C (13.8–22.4%), and 38°C (15.3–17.6%). At 45 and 12°C, the rates of acetate mineralized were lower than that observed at 25, 38, and 60°C, with average values of 10.7 ± 8.2 μM d^–1^ and 12.2 ± 6.9 μM d^–1^, respectively. At 12°C, the degree of mineralization was significantly lower than observed at 25–60°C (Table 1). Mineralization at 80°C was observed in only one of three replicates and to a small degree (53.8 μM or 5.38% acetate mineralized, Table 1) with a rate of 7.7 μM d^–1^.

**FIGURE 2:**
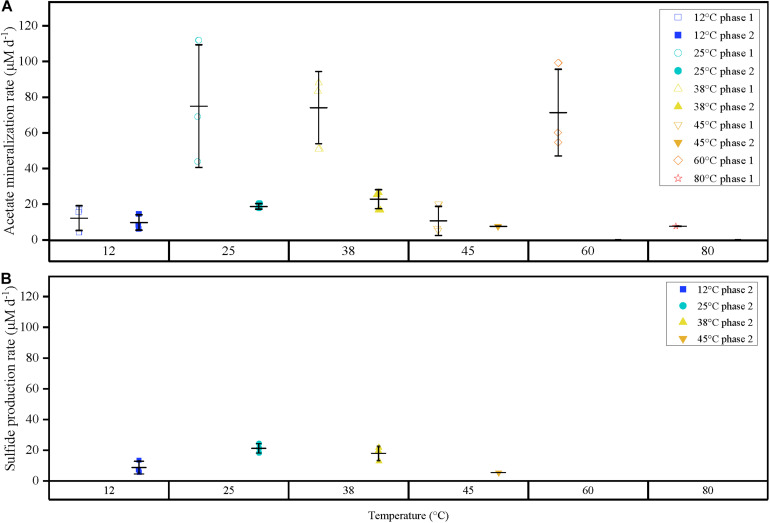
**(A)** Comparison of acetate mineralization rates for triplicates for both phases 1 and 2 represented as points differentiated by temperature (color), and phase (shape). **(B)** Sulfide production rates for phase 2 observed at 12, 25, 38 and 45°C-3. Outer horizontal bars indicate the standard deviation with respect to the triplicate average (where applicable). Acetate mineralization phase 2 was not observed at 60 and 80°C. Acetate mineralization at 45°C phase 2 and 80°C phase 1 is represented by a single point, respectively. Phase 1 is characterized by a short burst of activity compared to the prolonged activity in anaerobic phase 2.

**FIGURE 3:**
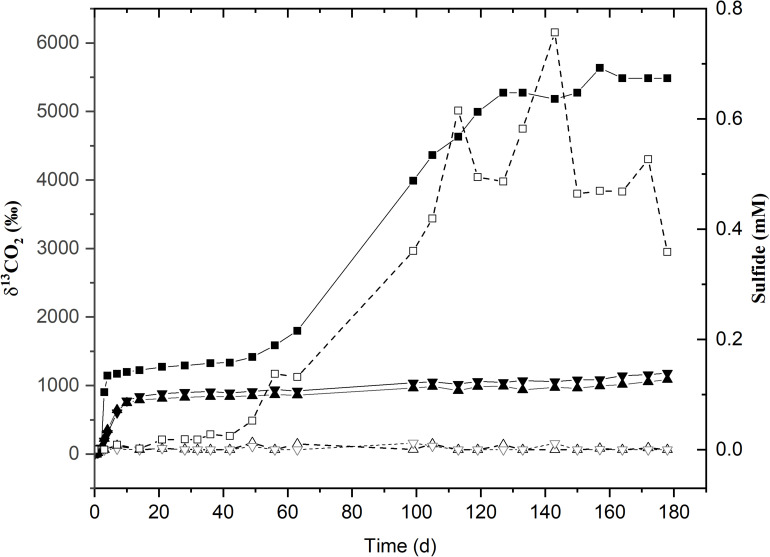
Time course for ^13^C-labeled CO_2_ production (solid lines with filled symbols) and sulfide (dotted lines with hollow symbols) incubated at 45°C. δ^13^CO_2_ and sulfide values for the sole active replicate (45°C-3) are represented by (■) and (□) respectively. (▲) and (▼) represent the δ^13^CO_2_ values for replicates 45°C-1 and 45°C-2. The remaining replicates for sulfide are given as (Δ) and (∇).

The second acetate mineralization phase (phase 2) was characterized by sulfide production; this started after a certain period of no δ^13^CO_2_ increment (plateau phase) which indicated the end of the first mineralization phase (Figures 1A,B). For triplicate microcosms incubated at 12, 25, and 38°C, the second mineralization phase began after around 10–14 days (25 and 38°C) and 63 days (12°C), respectively (Figure 1). The acetate mineralization rate in phase 2 was lower at 12°C (9.8 ± 4.3 μM d^–1^) when compared to 25 and 38°C (18.8 ± 1.5 μM d^–1^ and 22.9 ± 5.3 μM d^–1^ respectively) (Table 1 and Figure 2A). Acetate mineralization was completed in less than 60 days at 25 and 38°C, surpassing δ^13^CO_2_ values of 6000‰; whereas at 12°C, mineralization took longer and was completed within 214 days for all replicates (note that the curve in Figure 1A only represents the averaged value of the replicates and therefore only shows mineralization data up to 178 days). Sulfide production rates mirrored trends to that of acetate mineralization with the slowest rate at 12°C (8.8 ± 4.1 μM d^–1^), whereas higher rates were observed at 25 and 38°C (21.3 ± 3.1 μM d^–1^ and 18.0 ± 4.7 μM d^–1^ respectively) (Figure 2B). At 45°C, the biphasic behavior was found in only one replicate, 45°C-3, with concomitant sulfide production at a rate of 5.4 μM d^–1^ (Figure 3). No sulfide was produced in two of three replicates at 45°C as well as in all replicates at 60 and 80°C, corresponding with absent acetate mineralization; hence, phase 2 of acetate mineralization was absent in these replicates. Sterile controls did not produce any ^13^C-enriched CO_2_ or sulfide. The molar ratio of average sulfide produced to acetate mineralized in phase 2 were 0.89 (12°C), 1.01 (25°C), 0.86 (38°C), and 0.71 (45°C-3); thus slightly less sulfide was produced relative to the observed acetate mineralized in three of four temperatures (Table 2). The molar ratios were tested for normality using the Shapiro–Wilk test and found to be normally distributed (*P* > 0.05). The one-way ANOVA suggests that there were no significant differences between the molar ratios, *F*(1,8) = 1.6, *P* > 0.05.

**TABLE 2:** Acetate mineralization, sulfide production, and the ratio of sulfide produced to acetate mineralized at 12, 25, 38, and 45°C.

**Temperature**	**12°C**	**25°C**	**38°C**	**45°C**
Number of replicates	3	3	3	1
Acetate mineralized	913.1 ± 40	733.8 ± 58.8	731.9 ± 170.5	647.1
Sulfide produced	811.4 ± 74.6	744.2 ± 107	631.3 ± 163.6	461.8
$\frac{\mathrm{Sulfide}\,\mathrm{produced}}{\mathrm{Acetate}\,\mathrm{mineralized}}$	0.89	1.01	0.86	0.71

### Community Compositions at Different Temperatures

**FIGURE 4:**
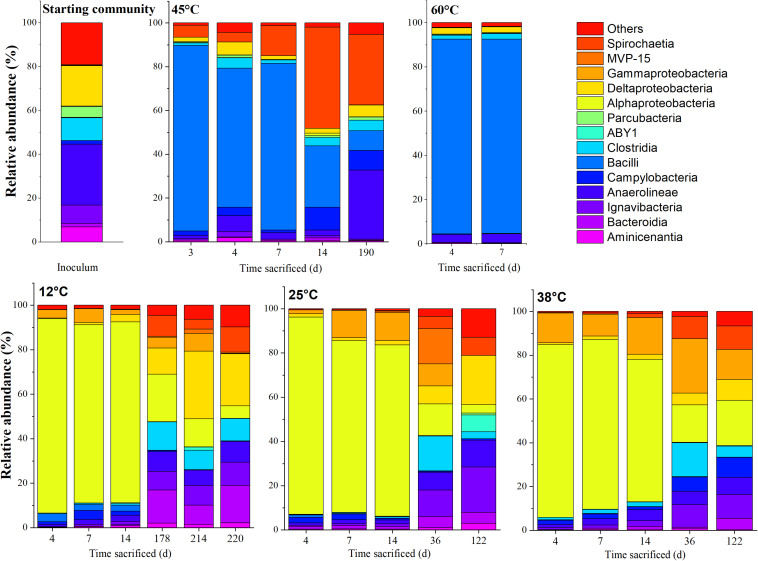
Time-resolved distribution of the bacterial community composition of the community assigned to order. Members which constitute < 5.0% across all samples were grouped as others.

**FIGURE 5:**
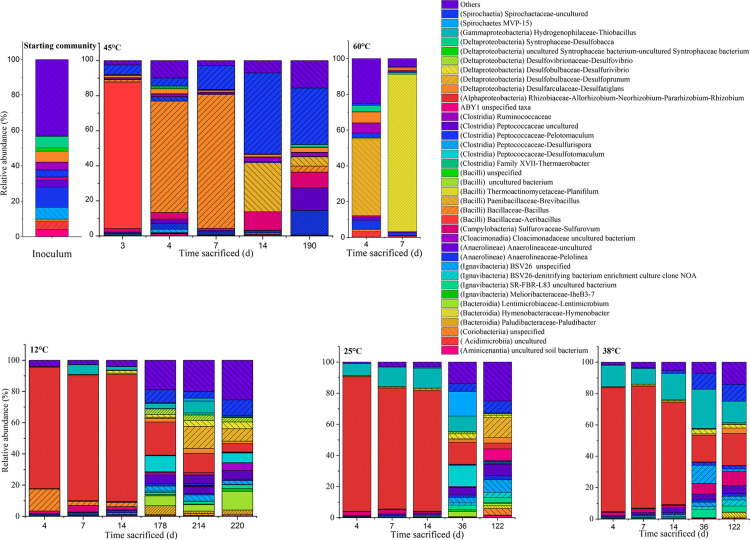
Time-resolved distribution of the bacterial community composition assigned to genera. Members which constitute < 3.0% across all samples were grouped as others (<3.0%). The class assignments of the respective genus assigned within the parentheses.

The community compositions at various stages of incubation for the respective temperatures, represented at the class and genus level, are shown in Figures 4, 5, respectively. The communities from the microcosms incubated at 80°C could not be sequenced due to insufficient amounts of DNA extracted from the biomass.

**FIGURE 6:**
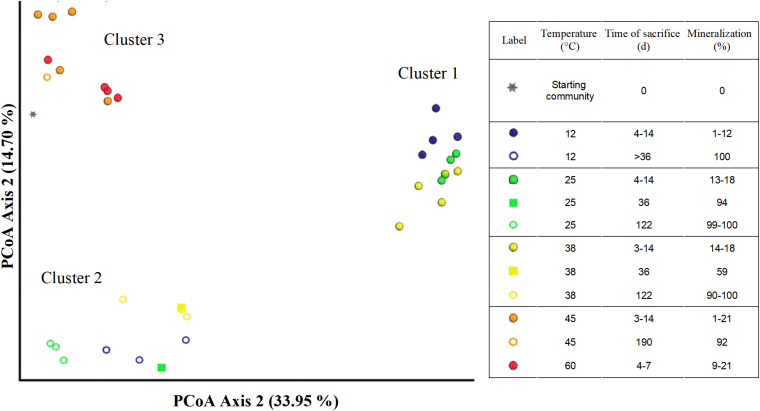
Principal coordinate plots (Bray–Curtis) of microbial communities in microcosms from different treatments and time points of sacrifice.

The MiSeq sequencing data returned 2,446,862 16S rRNA gene sequence reads with an average number of reads of 67,968 (±21,400) per sample and minimum and maximum sequence reads per sample of 21,993 and 118,715, respectively. The rarefaction curve showed variations in the number of observed amplicon sequence variants (ASVs), varying between 50 and 450 (Supplementary Figure S1). The total number of sequences per sample were used to represent microbial community composition (Figures 4, 5). However, for Bray-Curtis analysis (Figure 6), sequences of each respective sample were rarefied to the minimum of 21,993 sequences reads. The DNA extracted from the coarse sand used to set up the microcosms showed a relatively higher ASV richness, decreasing with the start of the experiment (Supplementary Figure S1).

In the inoculum, dominant phylotypes belonging to Anaerolineae (Chloroflexi) and Deltaproteobacteria (Proteobacteria) comprised approximately 24 and 17% of the assigned reads, respectively. Members of the Ignavibacteria (Bacteroidetes) and Clostridia (Firmicutes) were also amongst the main ASVs present, comprising 8 and 9%, respectively. On the genus level, abundant ASVs corresponded to the Anaerolinea-like *Pelolinea* (∼11.3%), as well as several other members of the Anaerolineae (∼12%), *Desulfobacca* (∼6%), *Desulfatiglans* (∼6%) within the Deltaproteobacteria, and an unspecified member of the Ignavibacteria related to the family BSV26 (7%).

#### Community Composition at 12, 25, and 38°C

Upon acetate mineralization in the first phase (Figure 1), microcosms at 12, 25, and 38°C sacrificed on days, 4, 7, and 14 were dominated by sequences affiliated to the mainly aerobic *Allorhizobium-Neorhizobium-Pararhizobium-Rhizobium* group belonging to the Alphaproteobacteria (∼65–86%) which were hardly detectable in the inoculum (0.16%). Other more abundant taxa in the first mineralization phase at 12°C to 38°C were phylotypes belonging to *Thiobacillus* (Gammaproteobacteria) (up to 16.8% compared to 0.06% in the inoculum) and *Sulfurovum* (Campylobacteria) (up to 4.1% compared to 1.6% in the inoculum). Conversely, phylotypes affiliated to *Bacillus* were more evident at 12°C (2.6–14.0%) than at 25°C (<0.3%) and 38°C (<0.3%) during the first mineralization phase.

In the second mineralization phase characterized by sulfide production (Figure 1), replicates at 25 and 38°C were sacrificed within 122 incubation days except for one replicate at 45°C, and triplicates at 12°C. The 12°C replicates were sacrificed on days 178, 214, and 220; the latter two replicates were sacrificed beyond the experimental duration and received an additional uniformly labeled acetate (∼1 mM) on day 178 to in order to maintain the culture until sacrifice. In spite of the different treatment, the three replicates were comparatively similar in abundance at both class and genus level. At the class level, the 12°C triplicates revealed several similarities to setups at 25 and 38°C sacrificed on days 36 and 122. Mainly, the abundant Alphaproteobacteria observed in phase 1 decreased from ∼65–86% to ∼3–21% at 12, 25, and 38°C, succeeded by a mixed community which varied amongst the three temperatures, generally represented by ASVs affiliated to the classes Ignavibacteria (Bacteroidetes), Anaerolineae (Chloroflexi), Deltaproteobacteria, Spirochaetia and Gammaproteobacteria. At 12°C, Deltaproteobacteria (10–28%) and Bacteroidia (7–16%) were the most abundant phylotypes (on days 178, 214, and 220), while at 25°C and 38°C, Ignavibacteria and Gammaproteobacteria were more abundant (on days 36 and 122). At 25°C, abundance of Ignavibacteria (10–18%), Gamma- and Deltaproteobacteria (8–21%) were comparable to that at 38°C with abundances of 10% and 5–8%, respectively.

At the genus level, in the 12°C replicates several sulfate-reducing genera belonging to the Deltaproteobacteria were more abundant: *Desulfoprunum* (0.8–14.0%), *Desulfurivibrio* (1.8–4.4%), *Desulfovibrio* (2.8–3.5%), and *Desulfatiglans* (1.5–3.2%). Other genera found to be enriched are the Bacteroidia-like *Lentimicrobium* (∼4.1–11.9%) and an uncultured member of the Spirochaetaceae (∼4.3–10.1%). Additionally, phylotypes affiliated to *Desulfotomaculum* (up to ∼10.0%), *Paludibacter* (1.5–5.6%) and an uncultured Anaerolineaceae member (4.3–5.6%) became apparent when conditions became sulfidic at the onset of the second phase.

At 25°C, in the second phase (day 122), sequences affiliated to the genera *Desulfoprunum* (12–14%), and an uncultured Spirochaetaceae (∼8%) were amongst the most abundant. Notably, members of *Thiobacillus* and *Sulfurovum* decreased from ∼7.5–12.7% (phase 1) to 0–7.6% (phase 2) and from ∼2.4% (phase 1) to 0.4–0.6% (phase 2), respectively.

At 38°C, the genera *Sulfurovum* (9.1%), *Desulfatiglans* (3.4%), an uncultured bacterium related to the SR-FBR-L83 family (∼5%), and Anaerolineaceae (5–12%) were some of the most abundant phylotypes. *Thiobacillus* sequences were the most abundant by day 36 (25%) but gradually decreased with time to ∼14% on day 122. Besides that, members of the Ignavibacteria (e.g., uncultured member of BSV26), Anaerolinea (e.g., *Pelolinea*), Deltaproteobacteria (e.g., *Desulfovibrio*), and Spirochaetia also experienced an uptick in abundance.

Besides the mentioned similarities in community compositions of the second phase at temperatures of 12, 25, and 38°C, some differences were observed. For example, abundances of *Desulfoprunum* members were higher at 12 and 25°C (up to 14.8%), than at 38°C (<3%), albeit after longer incubation. Members of the Ignavibacteria (e.g., belonging to the family BSV26) were most abundant at 25°C followed by 38 and 12°C. Lastly, *Lentimicrobium* abundances decreased from 12 to 25°C and were negligible in all replicates at 38°C and above.

#### Community Compositions at 45 and 60°C

At 45°C, the community composition was markedly different to that observed at 12, 25, and 38°C. Most replicates showed only one period of active mineralization, which occurred within the first 7 days of incubation. For the days 4 and 7 communities, dominant phylotypes were related to Bacilli, Campylobacteria, and Spirochaetia. Unlike phase 1 replicates at 12, 25, and 38°C, Alphaproteobacteria were not considerably enriched, ranging between ∼2–10% on days 3, 4, 7, and 14. Instead, several putatively mesophilic/thermophilic aerobes from the Bacilli lineage were present such as *Aeribacillus* (∼84%) on day 3, *Bacillus* (64–76%) on days 4 and 7 and *Brevibacillus* (∼28%) on day 14, indicating favorable temperature and electron acceptor condition. Furthermore, an uncultured Spirochaetia member (4–46%) and *Sulfurovum* (∼2–11%) were also present between days 3 and 14. The single replicate at 45°C (i.e., 45°C-3) which showed a second sulfidogenic phase was sacrificed after acetate was completely mineralized. The abundance of Bacilli was significantly lower (∼8.6%); specifically, sequences of the genera *Bacillus* (∼3%) and *Brevibacillus* (∼5%) which were less abundant when compared to the replicates on days 3–14. Instead, members belonging to Anaerolineae, Campylobacteria and Spirochaetiea emerged as the dominant class phylotypes. On the genus level, these are the *Pelolinea* (∼14%), uncultured Anaerolineaceae (∼13%), an uncultured Spirochaetaceae member (∼32%), and *Sulfurovum* (∼9%). *Desulfatiglans* had a relatively low but stable abundance throughout the 45°C incubations (∼1–3%), whereas other known sulfate-reducers were below 1%.

The samples incubated at 60°C which showed only one mineralization phase were sacrificed on days 4 and 7. At the class level, the community composition was dominated by Bacilli (∼32–88%). At the genera level, differences were observed amongst the replicates. Amongst the Bacilli, dominant genera were *Brevibacillus* (∼43%) on day 4 and *Planifilum* (∼89%) on day 7. In the day 4 replicate, *Pelolinea* (∼5%), *Desulfatiglans* (∼6%), *Desulfobacca* (∼3%) were also present but decreased at day 7 to low abundances (<1%).

### Succession of Communities Caused by Temperature

The Bray–Curtis Dissimilarity analysis of the microbial community data shows a distinct difference between temperatures and phases represented as three main clusters (Figure 6). The first cluster (indicated by filled blue, yellow or green spheres) represents communities incubated at 12, 25, or 38°C corresponding to the aerobic phase 1 (Table 1).

The second cluster (indicated by hollow blue, green, and yellow spheres) comprised microcosms incubated at 12, 25, and 38°C sacrificed after day 36 (denoted as > 36), and including day 36 (indicated by filled squares in green and yellow). Cluster 2 includes communities sacrificed at a later incubation time point at which considerably more acetate (53–94%) had been mineralized compared to the communities in cluster 1. The third cluster (indicated by filled or hollow orange or red spheres and black asterisks) is made of the microbial communities incubated at 45 and 60°C, and communities from the inoculum (Figure 1 and Table 1). Microcosms from 45°C (except replicate 45°C-3) and 60°C presented in this cluster were all sacrificed within the first 14 days, with a maximum mineralization of 21%. Additionally, this cluster also includes the sole active replicate 45°C-3, which achieved 98% mineralization after 190 days. The third cluster is also different from clusters 1 and 2. Community compositions between microcosms incubated at 12–25–38°C (cluster 1) were different from the inoculum (cluster 3), but the latter was more similar to the setups at 45–60°C (Cluster 3). Notably, communities at 45 and 60°C were likely different to those incubated at 12, 25, and 38°C. Secondly, two significantly different communities were established in the 12–25–38°C setting, belonging to phases 1 (cluster 1) and 2 (cluster 2) which suggests significant microbial community shifts with incubation times.

## Discussion

The investigated microbial community was originally enriched from a sulfidogenic, hydrocarbon-contaminated aquifer, and shown to be diverse and capable of degrading several aromatic hydrocarbons (Kleinsteuber et al., 2008; Herrmann et al., 2010; Taubert et al., 2012). Acetate was shown to be a metabolite upon sulfidogenic benzene mineralization, putatively metabolized by various phylogenetically different organisms (Rakoczy et al., 2011; Starke et al., 2016). We used this community as a model to determine the temperature range of anaerobic acetate mineralization and study the potential acetate-utilizing sulfate reducing degraders at different temperatures.

We postulate that mineralization occurred in two phases, a first aerobic phase and a second sulfidogenic phase. Aerobic mineralization is indicated by (i) the lack of sulfide (Supplementary Figure S2) or methane production from the reduction of sulfate and bicarbonate, the two principal electron acceptors present during the experimental set-up, and (ii) the bloom of typical aerobes affiliated to Alphaproteobacteria belonging to the genus *Allorhizobium-Neorhizobium-Pararhizobium-Rhizobium* (ANPR) (at 12–38°C) or Bacilli (at 45 and 60°C) (Figure 4). We rule out that nitrate or ferric iron might have been used as electron acceptors in this study, as both compounds were not amended. The coarse sand contains iron, but reduction of ferric iron has never been observed in former studies using this material (Vogt et al., 2007; Herrmann et al., 2008, 2010; Kleinsteuber et al., 2008; Taubert et al., 2012). Our data show a succession from oxic to anoxic conditions, illustrating conditions which usually occur in shallow subsurface systems and which could be promoted by ATES by introducing oxic heated or cooled water, thereby allowing us to compare mineralization rates of acetate under oxic and sulfate-reducing conditions in an altering microbial community. Given that acetate-degraders are ubiquitous since acetate occurs as a key intermediate in both aerobic/anoxic systems (Thauer et al., 1989), studying temperature or electron acceptor related shifts are therefore essential. Other studies focusing on communities’ behavior and successions at permanent anoxic conditions under different heat-change regimes are in progress (data not shown) and will be published separately. Approximately 0.2 mM acetate (equivalent to ∼20% of the amended amount) was consumed in the first phase, implying that up to 0.4 mM oxygen was supposedly available (Table 1). Under oxic conditions, acetate oxidation to carbon dioxide with oxygen (ΔG${}_{\text{r}}^{\circ }$ = −908 kJ mol^–1^) is far more exergonic, outcompeting other electron acceptor such as sulfate (ΔG${}_{\text{r}}^{\circ }$ = −63 kJ mol^–1^) (Thauer et al., 1989; Jesußek et al., 2013). However, once oxygen was depleted, the dominant aerobic phylotypes were replaced by communities consisting mainly of anaerobic phylotypes such as Deltaproteobacteria, Ignavibacteria and Anaerolineae.

The ANPR taxon encompasses a wide range of species (Berrada and Fikri-Benbrahim, 2014), aerobes or facultative anaerobes (mainly utilizing nitrates). Most phylotypes are mesophilic (Berrada and Fikri-Benbrahim, 2014), such as the recently isolated *R. terrae* sp. *nov*, exhibiting growth between 0–42°C (Ruan et al., 2020); accordingly, the immediate acetate-dependent growth of these putative aerobic phylotypes at 12, 25, and 38°C indicates a broad mesophilic temperature range of members of this genus. Besides ANPR, *Thiobacillus*-like members were also thriving in the initial aerobic phase at 12°C, 25°C, and especially at 38°C, likely from the aerobic oxidation of iron sulfides which have been previously identified in the used coarse sand (Vogt et al., 2007). These form black precipitates visible to the eye, and may be supported by acetate which is used as a carbon source by some *Thiobacilli* species (Robertson and Kuenen, 2006).

The Bacilli which became enriched in phase 1 at 45 and 60°C, belonging to the genera *Bacillus*, *Aeribacillus*, *Brevibacillus*, and *Planifilum* are affiliated to typical thermophiles. Some bacilli are capable of growth at 50°C (*B. subtilis*) and 65°C (*B. stearothermophilus*, range: 30–75°C), as well as on anaerobic agars at 50°C (e.g., *B. licheniformis*, *B. laterosporus*); and many can form resistant endospores (Warth, 1978; Slepecky and Hemphill, 2006). *Aeribacillus*, represented by only two species recently, *A. pallidus* and *A. composti*, has been described as thermophilic, aerobic, endospore-forming bacteria which can utilize acetate (Yasawong et al., 2011; Filippidou et al., 2015; Finore et al., 2017). *Brevibacillus* isolates (e.g., *B. borstelensis*) were reported to grow in the range of 40–70°C (Panda et al., 2014; Khalil et al., 2018). Also, *Planifilum* species were described as thermophiles (Hatayama et al., 2005; Zhang et al., 2007). Generally, formation of endospores allows survival at lower temperatures in the absence of oxygen; hence, the immediate endospore reactivation at higher temperatures explains the quick bloom of aerobic thermophiles at 45 and 60°C (Keynan et al., 1964; Berg and Sandine, 1970). The low observed mineralization of acetate at 80°C in one replicate is possibly due to weak activity of thermophiles thriving at maximal but lethal incubation temperature; indeed, the data indicate that hyperthermophilic phylotypes were absent or below detection limit in the microbial community investigated. These findings demonstrate a resilience of acetate-mineralizing aerobes over a temperature range of 12–60°C stemming from an inoculum which was held at anoxic, sulfidic conditions for several years and presumed to be dominated by obligate anaerobic phylotypes at the onset of the experiment (Figures 3, 4). Furthermore, the established aerobic communities are distinct between 12–38°C and 45–60°C.

Once oxygen was depleted, the second mineralization phase commenced following a period of lag phase and was more apparent (54–94% mineralized). Sulfide production peaked and stabilized between 0.8 and 1 mM for temperatures of 12, 25, and 38°C. Sulfide produced in Phase 2 was comparable to the theoretical sulfide production stoichiometry with respect to acetate mineralization, verifying that these two processes are connected (Table 2) and that sulfate was utilized as the terminal electron acceptor. The complete oxidation of 1 mM of acetate by dissimilatory sulfate reduction would yield 1 mM of sulfide and 2 mM of carbon dioxide (Eqn. 2). We calculated the ratio of sulfide produced to acetate mineralized for the second phase, discounting mineralization arising from the first aerobic phase (Table 2). Ratios of sulfide produced to acetate mineralized were < 1 at 12, 38, and 45°C-3, likely due to precipitation of FeS as previously described (Vogt et al., 2007; Herrmann et al., 2008; Kleinsteuber et al., 2008). Still, this relationship is within theoretical expectations (Kleikemper et al., 2002; Ozuolmez et al., 2015), including the value of 1.01 at 25°C (this study).

(2) $${\text{CH}}_{3}\,{\text{COO}}^{-}+{\text{SO}}_{4}^{2-}\to 2\,{\text{HCO}}_{3}^{-}+{\text{HS}}^{-}$$

In the 12–38°C setups, prolonged incubation saw putative sulfate reducing taxa becoming more evident including the genera *Desulfotomaculum* and *Desulfoprunum* (Figure 5), with the former seemingly more prevalent at 12 and 25°C, and not being represented at temperatures 38°C and above. *Desulfotomaculum* form endospores, and some species have been described for acetate oxidation (Widdel and Bak, 2006); psychrophilic *Desulfotomaculum* strains have been also previously reported (Klemps et al., 1985; Bahr et al., 2005; Aullo et al., 2013). Notably, mesophilic and moderately thermophilic *Desulfotomaculum* species were seemingly absent or below detection limit in our study although often described (Klemps et al., 1985; Scholten and Stams, 2000; Widdel and Bak, 2006; Aullo et al., 2013; Liu et al., 2018). For the genus *Desulfoprunum*, only a single species has been described so far, *D. benzolyticum*, a mesophilic freshwater isolate capable of acetate utilization (Junghare and Schink, 2015). While their study described an optimal growth of *D. benzolyticum* of 30°C with only little growth observed at 20 or 37°C, our results suggest that the *Desulfoprunum*-related phylotypes grown at 12°C are adapted to lower temperature ranges.

At 38°C, typical sulfate reducers also significantly increased in abundance in the sulfidic phase. For example, at day 36, these phylotypes comprised of *Desulfurispora* (11.6%) and *Desulfoprunum* (2.9%). Meanwhile, at day 122, *Desulfatiglans* (∼3.4%), *Desulfoprunum* (∼1.1%), *Desulforivibrio* (2.2%), and *Desulfurispora* (1.3%) were amongst the abundant sulfate reducers. It is worth to note that at day 36 acetate was still available, while these was completely utilized prior to sampling day 122, hence acetate unavailability could be a reason for the lower abundance, which could be attributed to other factors such as cell lysis or spore formation. The fact that *Desulfurispora* was highly abundant at day 36 hints that this organism metabolized acetate at 38°C. Notably, to our knowledge only one such species was described yet, growing at 40-67°C and unable to utilize acetate (Kaksonen et al., 2007). In contrast, the *Desulfurispora* phylotype seen in our study is abundant under mesophilic conditions, and as we did not detect any sulfate-reducing activity at 60°C. Lastly, *Desulfatiglans*, also detected in communities at 12 and 25°C have been reported for acetate oxidation under sulfate reducing conditions elsewhere (Jochum et al., 2018).

Besides the sulfate reducers, several other phylotypes also saw an uptick in abundance at 38°C. At day 36, phylotypes included an uncultured member of the Ignavibacteria (5.2%), *Sulfurovum* (6.5%), and uncultured member of the Anaerolineae (3.7%). Similarly, increased abundances were also reported at day 122, amongst them, an uncultured Anaerolinea member (5.1%), *Sulfurovum* (9.1%), three uncultured members of the Ignavibacteria (2.3–3.9%), which hinted at a possible role in acetate oxidation under mesophilic conditions. Ignavibacteria, with several members abundant in both 25 and 38°C setups, are not known for sulfate reduction, but were detected in various hydrocarbon-degrading microbial communities under various electron acceptor conditions including sulfate (Kummel et al., 2015; Westphal et al., 2017; Keller et al., 2018). However, their function in acetate metabolization at sulfate reducing conditions in our study is unclear. *Sulfurovum*, belonging to the Campylobacterales (Epsilonproteobacteria), was detected in several replicates at 38°C. Interestingly, Campylobacterales, have been reported as potential acetate consumers at sulfate-reducing conditions detected by protein-stable isotope probing studies using uniformly ^13^C-labeled acetate as substrate (Starke et al., 2016). In addition, Epsilonproteobacteria or specifically *Sulfurovum*-like phylotypes were enriched in hydrocarbon-degrading consortia (Kleinsteuber et al., 2008; Muller et al., 2009; Herrmann et al., 2010) which suggest that the *Sulfurovum* enriched in our 38°C setup could have consumed the acetate. While *Sulfurovum* was detected over a range of temperatures (22–66°C) in water-flooded oil reservoirs, they were not particularly enriched (Tian et al., 2017). However, in our study, *Sulfurovum* was particularly enriched at 38°C (up to 9%) and 45°C (up to ∼11%) while abundances were lower at 12°C (up to ∼4%) and 25°C (∼2%). In contrast, *Sulfurovum* abundance was negligible in almost all replicates at 60°C (<1%) which did not mineralize acetate at sulfate-reducing conditions. Hence, this Sulfurovum candidate might be active within the higher end of the mesophilic range (38–45°C).

One key result of our study is the observation that temperatures above 38°C (45°C, 60°C) strongly inhibited acetate mineralization at anoxic conditions, as only one replicate at 45°C could do so. Notably, none of the typical sulfate reducers that became enriched at 12–38°C seem to be abundant in this replicate, which raises the question of which phylotypes is actually responsible for sulfate reduction. The Spirochaetes, which increased to up to 32%, are often observed at anoxic hydrocarbon-contaminated sites; they are suggested to recycle necromass by fermentation, and have not been described to be directly involved in hydrocarbon degradation at anoxic conditions (Dong et al., 2018). Our data indicate that Spirochaetes are especially adapted to temperatures above the typical mesophilic range, which seem to be true as well for members of the Anaerolineaceae whose abundance increased to up to 27%. Anaerolineae were shown to comprise thermophilic members and were reported to reach high abundances during anaerobic digestion under methanogenic or sulfate reducing conditions, but due to a general lack of isolates and genomes their physiological capabilities are less well understood (Xia et al., 2016; Roy et al., 2018). Hence, it might be that these phylotypes become enriched by scavenging on dead biomass similar to the Spirochaetes. Although not yet described for dissimilatory sulfate reduction, we cannot rule out the possibility that some of them are able to do so. For instance, in a down-flow fluidized bed reactor operated at 25°C and mainly fed with acetate and sulfate, a community developed which comprised of 50% of Anaerolineaea and to a lesser extent of typical acetate-oxidizing sulfate reducers (Montoya et al., 2013). Recently, Anaerolineae phylotypes were shown to assimilate ^13^C-labeled acetate upon syntrophic acetate oxidation in a chemostat under methanogenic conditions (Yi et al., 2020); thus, it is also possible that acetate was syntrophically oxidized in our culture by Anaerolineae phylotypes, leaving the question open which organisms were actually responsible for sulfate reduction. Also 45°C lies within the overlap of the maxima end of the mesophilic range and the minima end of the thermophilic range, in the so called mesophilic-thermophilic boundary. Not much is known of bacteria tolerant in this boundary. Notably, in other studies, sulfide production was observed at higher temperatures compared to our study (Elsgaard et al., 1994; Bonte et al., 2013; Jesußek et al., 2013; Westphal et al., 2017).

Three distinct clusters in the Bray–Curtis confirmed the described shift in the microbial communities at 12, 25, and 38°C in phase 1 (cluster 1) to phase 2 (cluster 2), which are both markedly different from the inoculum (cluster 3). Community shifts are usually observed in environments shifting from oxic to anoxic conditions (Broman et al., 2017; Bush et al., 2017). Additionally, samples from replicates at 45 and 60°C in phase 1 contained sequences belonging to cluster 3 and were thus similar to the inoculum; the main difference between them is the emergence of putative aerobic species such as the Bacilli (Figure 5). The sulfidogenic replicate 45°C-3 also grouped in Cluster 3, although having a lower abundance of Bacilli-like phylotypes which were replaced by higher Anaerolineae and Spirochaetia abundances. The composition of cluster 3 of the Bray–Curtis analysis indicates that only a few members of the inoculum were actually active at temperatures ≥ 45°C. Notably, the results of our study are in contrast to those reported by Westphal et al. (2017) who observed acetate mineralization under both oxic and sulfate-reducing conditions in aquifer sediment columns experiments at 10, 25, 40, and 70°C.

The temperature growth/biochemical reaction correlation (Davey, 1993) would imply that mineralization rates increase with temperature (Meier et al., 2005; EPA, 2017). Similarly, increased rates in organic matter mineralization between 25 and 50°C were also reported (Hossain et al., 2017). However, in our study, this was not necessarily the case. In the aerobic phase, two temperature maxima were observed, first at 25°C (75 ± 34.4 μM d^–1^) and second at 60°C (71.4 ± 24.3 μM d^–1^). Mineralization rates increased from 12 to 25°C, in agreement with increased mineralization from 6 to 28°C observed before (Sansone and Martens, 1982). However, rates at 38, 45, and 60°C did not fit with this relationship, given that rates at 25 and 38°C (74.1 ± 20.2 μM d^–1^) were similar; and a rate minimum was observed at 45°C, being about six times lower than the values at 25 and 38°C. In the anaerobic phase, considering only replicates at 12–25–38°C, acetate mineralization rates (9.8 ± 4.3, 18.8 ± 1.5, 22.9 ± 5.3 μM d^–1^) matched the Arrhenius relationship, increasing with temperature (Table 1). The increased mineralization activity at 25 and 38°C compared to 12°C corroborates with prior studies on the enhanced biodegradation with increased temperatures (Jesußek et al., 2012; Griebler et al., 2016; Westphal et al., 2017). In contrast, the single sulfidogenic replicate 45°C-3, does not fit with the linearity observation, indicating non-optimal conditions for the acetate consumers at 45°C as microbial growth rates outside optimal conditions decrease with temperature until inhibition (Mohr and Krawiec, 1980; Okabe and Characklis, 1992; Huang et al., 2011). Inhibition of degradation processes at temperatures above 45°C has also been shown in other studies, e.g., for dechlorination (Deeb and Alvarez-Cohen, 1999; Friis et al., 2007b; Fletcher et al., 2011). Clearly, more studies are needed using different types of sediments to derive general assumptions on the physiological capacities of indigenous microbial communities at different, especially high (T ≥ 45°C) temperatures; inhibition temperatures might be experiment- or site-specific.

### Implications and Outlook

Our study highlights the key role of temperature toward microbial degradation at both oxic and strictly anoxic conditions. Albeit limited to laboratory batch microcosms, our study provides data on fundamental microbial community shifts and activities which help to evaluate related processes in real ATES systems. Our data emphasize the importance of the interplay of electron acceptor presence and temperature toward the activities of single members of the microbial community, and provides indications for possible temperature limits at distinct redox conditions. From the standpoint of ATES in temperate zones, with ambient groundwater temperatures of ca. 8–14°C, rates are expected to be higher with increasing temperature. At temperatures up to 38°C, which is beyond that of the typical ATES temperature threshold of 25°C in many European countries, sustained mineralization rates were observed at both oxic and anoxic conditions. However, the ultimate aim remains to assess the feasibility of HT-ATES, operating at temperature conditions only favorable for thermophilic or hyperthermophilic species. Meanwhile, the mesophilic-thermophilic boundary, such as 45°C could be an interesting starting point for future studies when trying to combine HT-ATES and *in situ* bioremediation, by identifying and characterizing mesophilic/thermophilic species capable of contaminant degradation. With observed sulfide production at 45°C, the feasibility for such reactions to occur at this temperature remains open; clearly more studies are required to understand the community resilience in the mesophilic-thermophilic boundary, using sediments of different origin and history.

In this study, temperature thresholds for aerobic mineralization are found to be higher compared to mineralization at sulfate-reducing conditions. This becomes the main caveat in the subsurface that become rapidly void of oxygen, particularly in contaminated zones (EPA, 2013). While aerobic mineralization is feasible (up to 60°C-this study) this cannot be assumed for anoxic condition. Nevertheless, HT-ATES could largely benefit from the twofer outcomes that allow higher thermal energy storage whilst simultaneously remediating contaminants without compromising anaerobic mineralization processes and rates. The uncertainty on how the combined heat storage and bioremediation approach could be achieved at elevated temperatures, i.e., >45°C, in bioremediation of hydrocarbon-contaminated aquifers still needs to be addressed. Additionally, seasonal ATES operations would drive subsurface temperature fluctuations (Saito et al., 2016), more so in HT-ATES. A deeper understanding of the effects of thermal energy storage and extraction toward mineralization and microbial community is an important step toward the goal of raising thermal storage thresholds. Community resilience and thermal cycling fatigue could also threaten ATES performance. Heat cycling experiments, by combining different heat exchange scenarios, together with stable isotope probing could elucidate the long-term effects of HT-ATES on ecosystem services and bioremediation potential. Furthermore, repopulation of HT-ATES from aquifer recharge or bioaugmentation could sustain or enhance bioremediation at elevated temperatures (Friis et al., 2007a,c).

### Summary

A microbial community adapted to sulfate reduction and hydrocarbon degradation amended with acetate showed distinct temperature-related effects on mineralization and sulfide production pattern observed at temperatures up to 38°C. Sulfide production was notably absent at 60°C and 80°C, and in two of three replicates at 45°C. Biphasic mineralization phases at 12°C to 38°C and a single phase mineralization at 60°C illustrated the consecutive use of oxygen and sulfate as electron acceptors at 12–38°C and the exclusive use of oxygen as acceptor at 60°C. At 80°C, aerobic mineralization was likely impaired while mineralization under sulfate reduction was absent, likely from the absence of thermophiles. Microbial community analysis as well as Bray–Curtis dissimilarity further highlights the role of temperature in community differences under aerobic and sulfate-reducing environments. The discovery of aerobic Alphaproteobacteria from an anaerobically maintained inoculum, suggests a notable resilience of these aerobes living under prolonged anoxic conditions. Meanwhile, Bacilli detected at the mesophilic/thermophilic range would be of no surprise owing to its endospore-forming ability. Aerobic acetate mineralization rates were comparable at 25, 38, and 60°C but about six to seven folds higher than at 12 and 45°C. Anaerobically, acetate mineralization rates for 12, 25, and 38°C increased with temperature, with stoichiometric production of sulfide, however, the lowest rate was observed in the sole sulfide producing replicate at 45°C. Microbial community analysis only found several typical sulfate reducers such as *Desulfoprunum* with varying abundances at 12 to 38°C. In contrast, no typical sulfate-reducers were significantly stimulated in the sulfidogenic 45°C replicate; instead Anaerolinea and Spirochaetia members were more dominant. Herein, this study found that temperatures up to 38°C does not seem detrimental to the removal of acetate, but a higher temperature (>45°C) remains a limitation where remediation strategies are concerned. Nevertheless, this proposes a potential new discussion for sulfate reduction at the mesophilic-thermophilic boundaries especially if HT-ATES proved unfeasible. This study demonstrates how the community could adapt to temperature and electron acceptor availability. More importantly, temperature could influence mineralization rates, as well the potential actors involved in degradation.

## Data Availability Statement

The datasets presented in this study can be found in online repositories. The names of the repository/repositories and accession number(s) can be found below: https://www.ebi.ac.uk/ena, PRJEB40338.

## Author Contributions

MB planned and performed the experiments, analyzed the data, and wrote the manuscript. CV planned the experiments and edited the manuscript. HR edited the manuscript. All the authors contributed to the article and approved the submitted version.

## Conflict of Interest

The authors declare that the research was conducted in the absence of any commercial or financial relationships that could be construed as a potential conflict of interest.
