# Supplementary material for: Effect of Temperature on Acetate Mineralization Kinetics and Microbial Community Composition in a Hydrocarbon-Affected Microbial Community During a Shift From Oxic to Sulfidogenic Conditions
Source: Front Microbiol. 2020 Dec 17;11:606565. doi: 10.3389/fmicb.2020.606565 (PMC7773710; doi:10.3389/fmicb.2020.606565)
Supplement: Supplementary file 1 [file Image_1.pdf]

Supplementary Figure S1

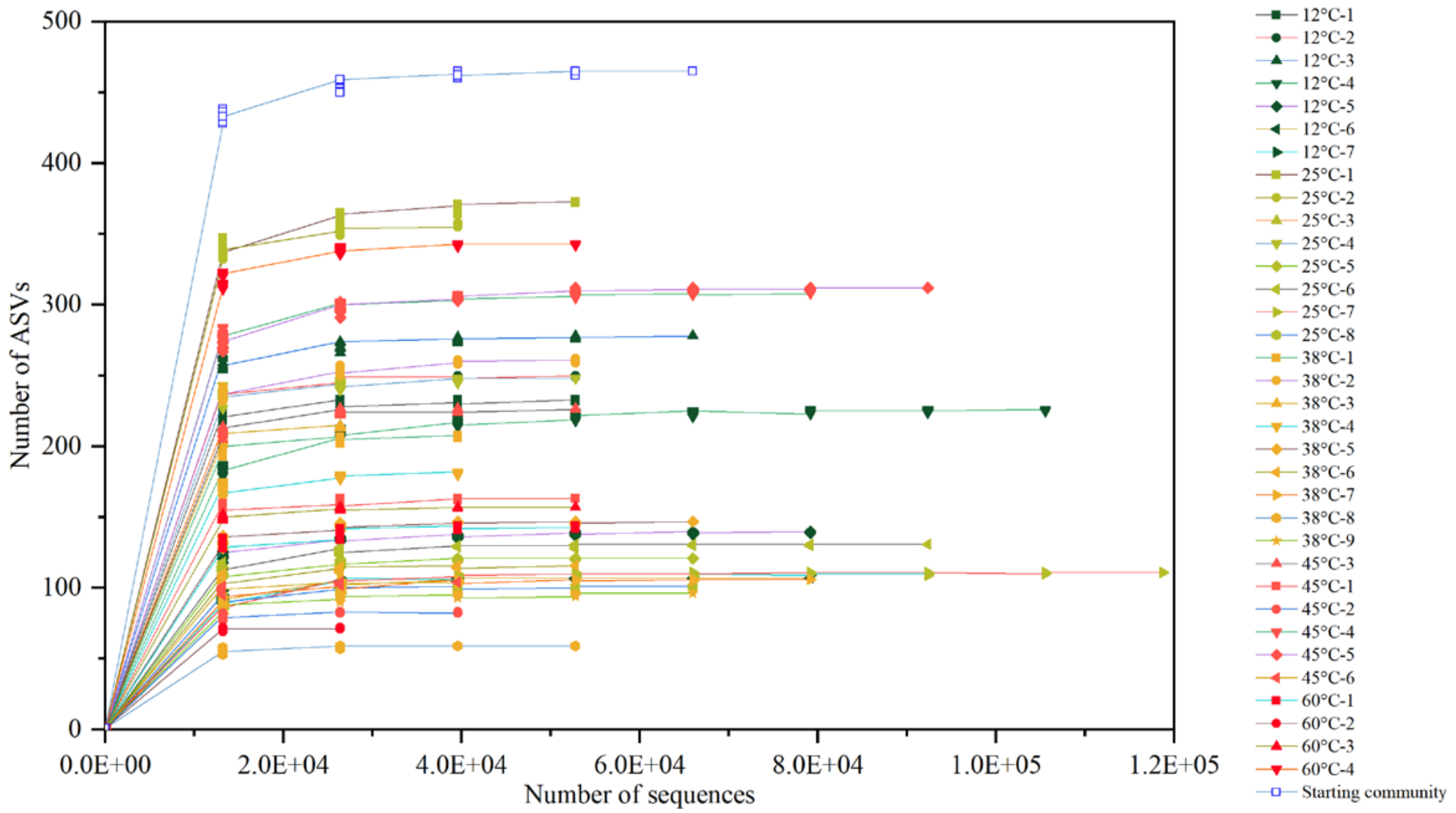

Replicate	Number of ASVs	Replicate	Number of ASVs	Replicate	Number of ASVs	Replicate	Number of ASVs	Replicate	Number of ASVs
12°C-1	233	25°C-1	373	38°C-1	208	45°C-1	227	60°C-1	143
12°C-2	250	25°C-2	358	38°C-2	262	45°C-2	163	60°C-2	72
12°C-3	278	25°C-3	212	38°C-3	215	45°C-3	83	60°C-3	157
12°C-4	226	25°C-4	248	38°C-4	180	45°C-4	308	60°C-4	342
12°C-5	140	25°C-5	121	38°C-5	147	45°C-5	312		
12°C-6	107	25°C-6	131	38°C-6	116	45°C-6	104		
12°C-7	110	25°C-7	111	38°C-7	106				
Starting community	465	25°C-8	101	38°C-8	59				
				38°C-9	96				

Supplementary Figure S1| Rarefaction curves of replicate microcosms at each incubation temperature including number of ASVs, before being rarefied to the minimum sequence reads.
