# Supplementary material for: Effect of Temperature on Acetate Mineralization Kinetics and Microbial Community Composition in a Hydrocarbon-Affected Microbial Community During a Shift From Oxic to Sulfidogenic Conditions
Source: Front Microbiol. 2020 Dec 17;11:606565. doi: 10.3389/fmicb.2020.606565 (PMC7773710; doi:10.3389/fmicb.2020.606565)
Supplement: Supplementary file 2 [file Image_2.pdf]

Supplementary Figure S2

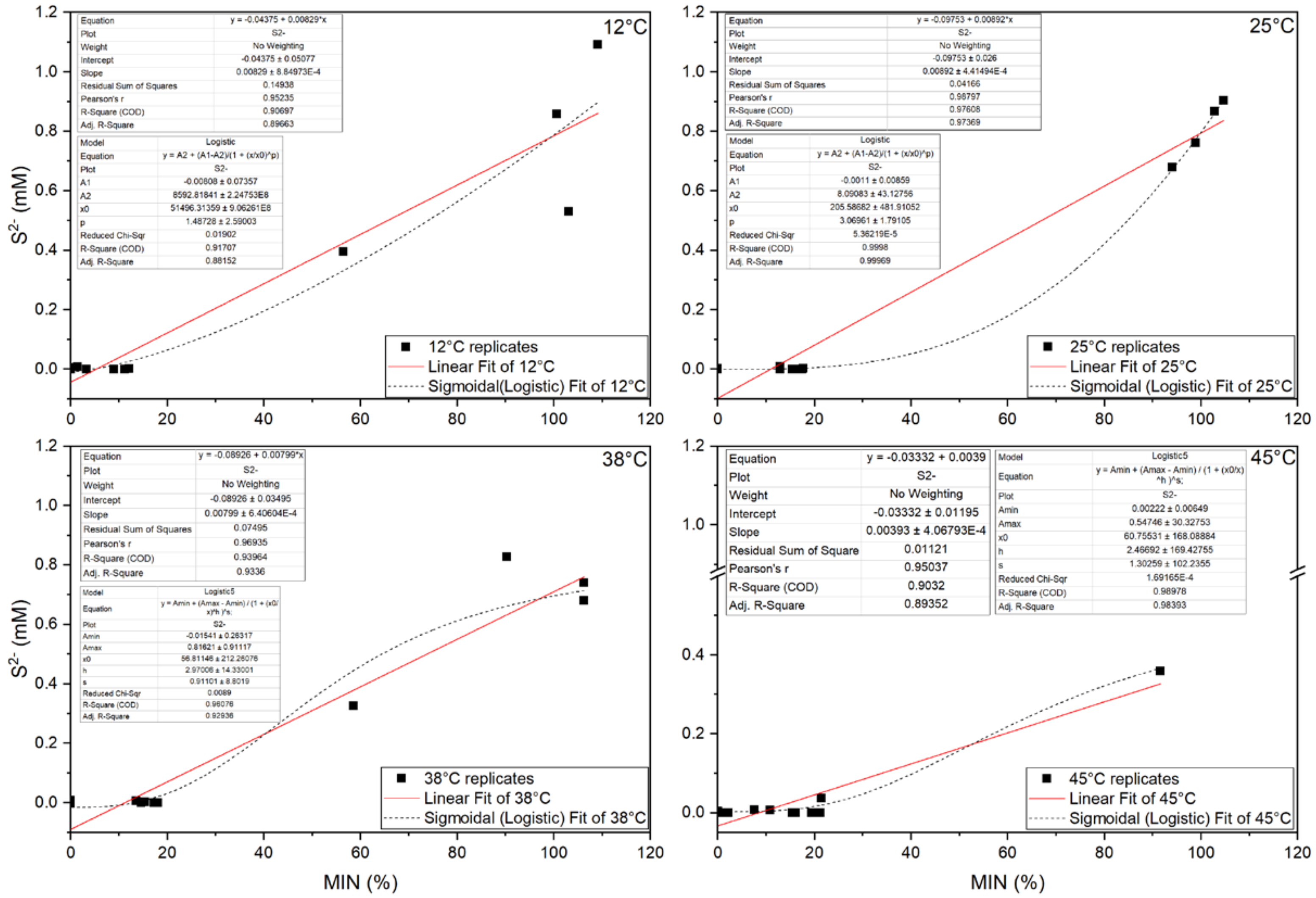

Supplementary Figure S2| Scatter plots of percent mineralization (MIN) for each replicate vs sulfide concentration for temperatures 12°C, 25°C, 38°C and 45°C. The data were fitted with a linear fit (–) and a corresponding sigmoidal (Logistic/ Logistic5) Fit to predict points during acetate mineralization when sulfide production commences.
