# Supplementary material for: Effect of Temperature on Acetate Mineralization Kinetics and Microbial Community Composition in a Hydrocarbon-Affected Microbial Community During a Shift From Oxic to Sulfidogenic Conditions
Source: Front Microbiol. 2020 Dec 17;11:606565. doi: 10.3389/fmicb.2020.606565 (PMC7773710; doi:10.3389/fmicb.2020.606565)
Supplement: Supplementary file 3 [file Image_3.pdf]

Supplementary Figure S3

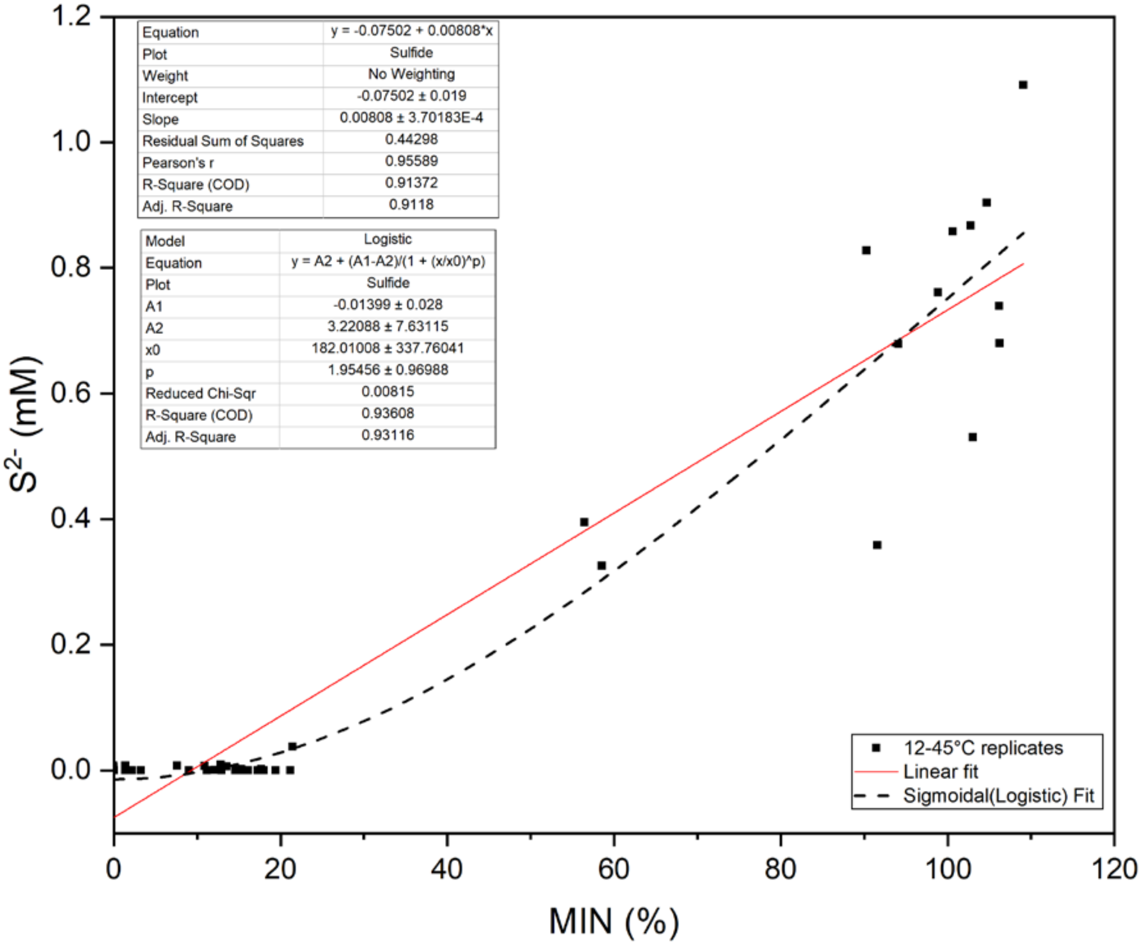

Supplementary Figure S3| Scatter plot of mineralization percent of acetate (MIN) vs sulfide concentration of replicates incubated at 12-45 °C. Replicates at 60°C and 80°C were not included at there was no significant sulfide production observed in any of the replicates. Linear fit (—) and sigmoidal fit (- - -) shown to represent predict trends of the observations. A linear fit would suggest that sulfide was produced observed throughout mineralization). The sigmoidal fit would suggests the likely presence of another electron acceptor present utilized during the first ~20% of acetate mineralization prior to the start of sulfide production.
