# Supplementary material for: Effect of Temperature on Acetate Mineralization Kinetics and Microbial Community Composition in a Hydrocarbon-Affected Microbial Community During a Shift From Oxic to Sulfidogenic Conditions
Source: Front Microbiol. 2020 Dec 17;11:606565. doi: 10.3389/fmicb.2020.606565 (PMC7773710; doi:10.3389/fmicb.2020.606565)
Supplement: Supplementary file 4 [file Image_4.pdf]

Supplementary Table S1| Recipe of the modified mineral salt medium (Bak and Pfennig, 1991)

	Component	Description
Basal medium	KH_2PO_4	0.2 g
	NH_4Cl	0.1 g
	KCl	0.5 g
	$CaCl_2$	0.1 g
	$MgCl_2$	0.2 g
	Na_2SO_4	1.42 g
Components were dissolved in ca. 945 mL distilled water, autoclaved (121°C, 20 min) and purged with N_2 afterwards		
The following anoxic stock solutions were added to the Basal medium in the glove box:		
Stock solutions	Volume added	Description
$NaHCO_3$	30 mL	CO_2-saturated 1 M $NaHCO_3$ solution
Vitamin solution	5 mL	Component (mg per L):<br>8 4-Aminobenzoic acid<br>2 D(+)-biotin<br>20 Nicotinic acid<br>10 Ca-D(+)-pantothenate<br>30 Pyridoxamine hydrochloride<br>20 Thiamine dichloride<br><br>Solution was filtered (0.2 µM) and purged with N_2 afterwards, stored subsequently in the dark at 4°C
Trace element solution SL-10 (free of EDTA)	1 mL	Component (per L):<br>8.5 mL HCl (37%)<br>1.5 g $FeCl_2$ x 4 H_2O<br>6 mg H_3BO_3<br>190 mg $CoCl_2$ x 6 H_2O<br>100 mg $MnCl_2$ x 4 H_2O<br>70 mg $ZnCl_2$<br>36 mg Na_2MoO_4 x 2 H_2O<br>24 mg $NiCl_2$ x 6 H_2O<br>2 mg $CuCl_2$ x 2 H_2O<br><br>Solution was filtered (0.2 µM) and purged with N_2 afterwards, stored subsequently in the dark at 4°C
Vitamin B_{12} solution	1 mL	Component (per L):<br>0.05 g Cyanocobalamin<br><br>Solution was filtered (0.2 µM) and purged with N_2 afterwards, stored subsequently in the dark at 4°C
Selenite-tungsten solution	1 mL	Component (per L):<br>0.5 g NaOH<br>3 mg Na_2SeO_3 x 5 H_2O<br>4 mg Na_2WO_4 x 2 H_2O<br><br>Solution was filtered (0.2 µM) and purged with N_2 afterwards, stored subsequently in the dark at 4°C
HCl (2 M)	ca. 2 mL	adjust subsequently to pH 7
Sodium dithionite	1 mL	100 mM stock solution
Autoclaved, N_2 purged distilled water was added to top up media to 1 L		
